# Economic policy and public health: Insights from the history of the *Canadian Journal of Public Health*

**DOI:** 10.17269/s41997-024-00940-3

**Published:** 2024-10-22

**Authors:** Lindsay McLaren, Eric Mykhalovskiy

**Affiliations:** 1 University of Calgary, Calgary, AB Canada; 2 York University, Toronto, ON Canada

**Keywords:** Public health, History, Economics, Policy, Equity, Santé publique, histoire, économie, politique (principe), équité

## Abstract

The nearly 115-year history of the *Canadian Journal of Public Health* (CJPH) provides an important opportunity to reflect on and learn from our past. In response to an invitation to members of the CJPH Editorial Board to curate historical articles around a theme, we undertook a historical examination of our field’s engagement, as gleaned through the pages of CJPH, with economic policy. This was inspired by the now well-established connections among political economic policy, population well-being, and health equity. Our analysis of six historical volumes (1917, 1933, 1941, 1961, 1995, and 2013) led to three key findings. First, we found only a slim historical foundation for public health engagement with the economy overall. Second, we observed a strong and seemingly subconscious allegiance to dominant economic paradigms, despite their incompatibility with root causes of health inequities. Third, even though socio-economic inequalities in health are a long-standing preoccupation of CJPH authors, those inequalities are consistently and curiously divorced from their roots in political economic systems. Our findings provide a historical foundation for thinking about how our public health community could be encouraged to engage constructively towards an economic system that supports, rather than obstructs, population well-being and health equity.

## Abstract (fr)

À l’approche du 115^e^ anniversaire de la *Revue canadienne de santé publique* (RCSP), il est important de réfléchir à l’histoire de la Revue et d’en tirer des leçons. Répondant à l’invitation du comité éditorial d’organiser des articles du passé autour d’un même thème, nous avons mené, à la lumière des informations glanées dans les pages de la Revue, un examen historique de l’implication de la santé publique dans la politique économique. Nous nous sommes inspirés des liens aujourd’hui bien établis entre la politique économique, le bien-être des populations et l’équité en santé. Notre analyse de six volumes de la Revue (1917, 1933, 1941, 1961, 1995 et 2013) permet de tirer trois grands constats. Premièrement, l’implication de la santé publique dans l’économie en général semble reposer sur des bases ténues. Deuxièmement, nous avons observé une allégeance solide et apparemment inconsciente envers les paradigmes économiques dominants, en dépit de leur incompatibilité avec les causes profondes des iniquités en santé. Troisièmement, bien que les inégalités socioéconomiques en santé préoccupent depuis longtemps les auteurs de la RCSP, ces inégalités sont constamment et curieusement divorcées de leurs racines dans les systèmes politico-économiques. Nos constats donnent des assises historiques à une réflexion sur les moyens possibles d’encourager la communauté de la santé publique à se mobiliser utilement pour que le système économique favorise, plutôt que de les entraver, le bien-être des populations et l’équité en santé.

## Introduction

It is well established that population well-being and health equity are significantly influenced by the social determinants of health, including their public policy antecedents and ideological underpinnings (CSDH, 2008; Raphael et al., 2020). Economic policy advanced by governments plays a significant role in shaping health determinants and their distribution. Examples include government spending on public services and income supports; tax policy that shapes the distribution of income and wealth; financial and trade liberalization that affects the global distribution of income and wealth and causes profound shifts in labour markets, creating new employment for some and precarious employment for others; and regulations that affect extractive industries and ecosystems with implications for climate change and other aspects of environmental health (NCCDH, 2022; CPHA, 2015).

Despite the significance of economic policy for health, engagement by public health communities with economics tends to be narrow, focusing on, for example, return on investment of public health interventions (Masters et al., 2017) or proportion of health sector spending devoted to public health activities (Fiset-Laniel et al., 2020; McLaren & Dutton, 2020). Limited engagement with upstream determinants as they are embodied in economic policy represents a significant missed opportunity to strengthen conditions for population well-being and health equity, the stated goals of public health (CPHA, 2017).

Although economic policies offer a strong mechanism to strengthen population well-being and health equity, they have distinctly not been leveraged to that end in Canada or indeed in most of the world. The significant and highly inequitable impacts of recurring global financial crises, escalating global climate change and environmental degradation, and the COVID-19 pandemic leave no doubt about the incompatibility between neoliberal capitalism (the dominant global economic system since the early 1980s) and social and ecological determinants of health equity (Bump et al., 2021; Ruckert & Labonté, 2014). A new political economic paradigm is desperately needed. How might our public health community be encouraged to engage constructively towards an economic system that is supportive of population well-being and health equity, including economic policy advanced by governments and the broader political economic paradigm in which it is situated? To help answer this question, we turn to a historical examination of our field’s engagement with economic policy. Focusing specifically on the *Canadian Journal of Public Health* (CJPH), which has a long-standing focus on prevention and well-being,^1^ we consider the historical foundation for contemporary public health engagement with economic policy and look for lessons that might be learned from how past generations of public health actors recognized and responded to the connection between the economy and health.

We divided the history of the journal (1910–present)^2^ into six time periods corresponding to key economic circumstances or events. These periods were 1910–1928 (from the journal’s first issue through the first world war and its aftermath); 1929–1938 (the 1929 stock market crash and ensuing Great Depression); 1939–1945 (the economic preoccupation of the second world war); 1946–1979 (the Keynesian period); 1980–2007 (the early neoliberal period); and 2008–2021, which Osberg (2021) refers to as “zombie neoliberalism” because, while the credibility of neoliberalism has been shattered, an alternative paradigm has not yet emerged. Within each period, we randomly selected one volume (1 full year) and we each read it in full, focusing on context and key historical events, topics covered, type and range of authors, discussion of economic issues, and discussion of intervening in the economy in the interest of health (broadly defined). We then compared and revised our analytic notes for each selected volume. Based on their heuristic value, we selected specific papers,^3^ denoted below in ***bold italic font***, to showcase in full as part of this special section.

## 1917, Volume 8: Public health, the economy, and eugenics

The CJPH was quite a different journal in 1917, including in name. There was very little empirical research; the journal largely consisted of reports of conferences and meetings, as well as transcripts of local speeches or conference addresses. Addresses were delivered, almost without exception, by male physicians serving in public health officer roles in Canada or elsewhere—usually the United States or Great Britain. Advertisements in the journal suggested an early role in creating a market for certain goods and services, including international travel, medical devices, and cleaning products. Placing present-day concerns in historical perspective, Volume 8 included lamentations about misinformation (“quacks”), people not complying with public health regulations, and the public’s poor understanding and appreciation of public health.

Within the first world war context, there was some recognition of economic factors as root causes of illness, but in ways that were infused with sexism, racism, and classism. Graham (1917), for example, discussed the mental health consequences of “grinding toll and abject poverty”, which naturally leads workers to seek relief in things like alcohol. The war, Graham argued, gives people a sense of purpose and thus might be expected to reduce mental illness, especially among middle-class women as they discover that life is more than “the tepid gossip of a church sewing society”. Volume 8 also contained early indications of economic rationalizing, where attention to a health problem was justified based on its monetary cost. Wartime examples included concerns about the expense, due to the need for costly medical treatment, of venereal disease (Hastings, 1917) and of enlisting tuberculosis (TB)-infected soldiers (Hattie, 1917) (the influenza pandemic of 1918–1919 and the likely significant economic concerns it raised for the public health community had not yet occurred). Economic reasoning was also used to mobilize eugenic discourse: Knopf (1917), for example, argued for birth control to reduce the number of physically, mentally, and morally unfit children who are characterized as costly to care for and as detracting from the vigour of the population.^4^

There were a few examples in Volume 8 of the Canadian public health community engaging with economic policy or state mechanisms to address problems rooted in economic circumstances. McCullough’s (1917) paper, for example, was situated in the wartime context where owners of munitions factories were permitted to extend work hours to support the war effort. Although the paper did not mention “economy” or “economics”, it recommended a reduction in daily working hours, including on the grounds of health. In a paper promoting large family sizes, Weston (1917) argued for economic policies to help offset the financial cost of children. He specifically advocated for a tax on every household to raise and redistribute funds to support large families. A third example is a paper about housing by ***Cartlidge*** (1917), the Chief Sanitary Inspector in Moose Jaw, Saskatchewan. In the context of the anticipated post-war “tide of immigration”, Cartlidge argued for regulation around unscrupulous builders “whose chief purpose is cheap construction” to be offered at high rent. We were struck by the present-day relevance of this 1917 paper’s focus on the intersection of public health and power dynamics embedded within housing markets and urban development.

## 1933, Volume 24: Turning inward in a time of economic crisis

In the 1933 context of the Great Depression, the evident response of the CJPH community was to turn inward and hunker down. Rather than engage directly with the likely significant health implications of the economic circumstances, authors seemed to accept the depression as a limit that couldn’t be addressed in any significant way. Indeed, they were more likely to lament the depression’s consequences for CPHA membership and journal sponsorship than for the public’s health.

Consistent with an inward retreat, Volume 24 conveyed a prominent biomedical orientation. Many articles focused on medical or clinical aspects of specific diseases (e.g., TB, smallpox, measles) in a decontextualized way. In a discussion of diarrhoea and enteritis, for example, McKinnon (1933) discussed alternative medical diagnoses and flies as sources of bacterial infections, but offered no discussion of social or economic circumstances that cause people to be more exposed or vulnerable to disease vectors. These disease-specific pieces often took the form of epidemiologic reports, and indeed, by this time the journal had a clear emphasis on data and statistics, including a section dedicated to “Epidemiology and vital statistics”.

A good illustration of the nature of CJPH engagement with matters of the economy in 1933 was in articles on food and nutrition. Issue #5 featured several articles about how state relief programs might incorporate elements of nutrition. The paper by ***McHenry*** (1933) is emblematic of the dominant public health orientation at the time of helping people to adapt to the (implicitly fixed) economic circumstances by making better (more nutritious and prudent) decisions about the family diet. Bell (1933) likewise aimed to help people to respond to the economic and social calamity of the depression, by incorporating emerging nutrition science in prudent public health advice about food. Goodeve (1933) described food relief work in Montreal, which entailed educating mothers to make better decisions, lamenting “those unfortunate people who have never learned to spend their money in the most profitable way”. Such efforts were nearly always directed at mothers, thus illustrating the historical roots of the gendered, heteronormative, middle-class, and white bias of public health’s contemporary moral project.

Perhaps the closest thing to productive critical engagement with economic policy in 1933 occurred in writings about industrial and occupational health. An editorial in Issue #4, for example, lamented that hazards in the workplace are basically left to individual firms and factories to address as they see fit according to their business interests, while “legislation and inspection are left far behind” (Cunningham, 1933). While such comments hint at the need to establish limits on economic activity to protect workers, they stop short of the key insight that these political economic dynamics should be a greater preoccupation for a field concerned with the health of the public. Overall, the nature of CJPH engagement with economic policy in 1933 seemed to us, if anything, a step backwards compared to 1917.

## 1941, Volume 32: War, economy, and the health of the nation

Published during the second world war, there was some indication in Volume 32 of connections being drawn among the war, matters of the economy, and public health. In general, those connections revealed economic rationalization seen earlier, where the health of the nation was framed as an economic good (that is, necessary to support the industrial and military requirements of war) to which public health could contribute via economically efficient interventions and the mobilization of science, which by 1941 had a clear presence in the journal.

This narrative played out in a few ways. One, continuing a theme from 1933, was articles on the nutritional status of the population. Although inequities in nutrition by economic circumstances were evident and recognized to be caused by inadequate income, CJPH authors persisted in advancing a downstream, patronizing practice of helping people to be smarter and more parsimonious in their shopping and cooking (e.g., Hunter & Pett, 1941). A specific emphasis in 1941 was on milk, which was promoted in an almost propaganda-like manner as a “superfood”. Articles on various aspects of milk—including pasteurization, labelling, distribution, and consumption—illustrated public health’s acceptance of the economic status quo. For example, an article on compulsory pasteurization legislation in Ontario was praised for not hurting the dairy industry (Editorial, 1941, Issue #4). Emblematic of public health’s orientation, a paper by ***Kitching*** (1941) reported on a survey of milk consumption of “family units” in Vancouver. Finding, not surprisingly, that milk consumption was low and uneven across the population, it recommended education, including to “housewives” in lower-income families so that they would, as described in the issue’s editorial, “put first things first”.

A second way that intersections among the war, the economy, and public health played out was in articles on worker health and safety; and indeed, by this point, “Industrial Hygiene” was a named section of the journal. The health of workers was frequently expressed in economic terms (e.g., monetary costs of lost workdays) to justify its importance. Echoing an observation from 1917, authors in Volume 32 drew some thoughtful connections between work circumstances and health. An “Industrial Hygiene” abstract in Issue #2, for example, noted how elevated suicide rates among railroad employees related to “unstable business conditions”, which brought to mind recent political economy scholarship (e.g., Stuckler & Basu, 2013). In some cases, such connections manifested in attention to economic policy. An “Industrial Hygiene” abstract in Issue #10, for example, recommended—in light of overtime and overtired workers—a reduction in weekly hours of work by the ministry of labour.

Third, the war, economy, and health nexus of 1941 prompted some CJPH authors to argue for broad, large-scale redistribution of resources to improve health, especially for those living in poverty. McCann (1941), for example, called for massive extensions of the welfare state, recognizing that “sickness and poverty have always gone hand in hand but I believe poverty is the cause of more illness than the result of it … [T]he solution … is social and economic”. However, public health’s racist and classist moral project continued to rear its ugly head, such as in Dolman’s (1941) recommendation that tax benefits should be used to promote middle-class families to have more children to save the nation.

Although not new in 1941, we also noted that philanthropic and corporate funding had a considerable presence in Volume 32. This presence, which is not innocuous for a field concerned with the public’s health, included support of CPHA activities by the Rockefeller Foundation, the Canadian Life Insurance Officers Association, the Metropolitan Life Insurance Company, and the Kellogg Foundation (Issue #7).

## 1961, Volume 52: The welfare state, efficiency, and accountability

The Cold War context of Volume 52 manifested most obviously in attention to “new” public health concerns of nuclear war, biological/chemical weapons, and radiation fallout. Despite clear links to global political economy, notably the Cold War competition between the USSR’s (Union of Soviet Socialist Republics) state-planned and the US-led liberal market models, these problems were generally discussed from a clinical or biomedical perspective. Chronic diseases had emerged by 1961 as a preoccupation for the CJPH community, and with that emergence came some limited engagement with matters of the economy; for example, lamentation about “economic forces” (unspecified, but likely referring to the economic and political power of the tobacco industry) that thwarted lung cancer prevention efforts. Although the journal continued to include transcripts of conference speeches, Volume 52 contained an increasing proportion of research papers, including some French-language papers. This was in the context of the 1960 establishment of the Medical Research Council of Canada, the precursor to the Canadian Institutes of Health Research (Issue #2).

With respect to public health and economic policy, we had two key observations from Volume 52. The first is around what appeared to be an uneasy relationship between public health and welfare services. This was in the context of the emerging welfare state, which itself reflected the social and economic hardships of the Great Depression, the clear need for state-sponsored welfare systems to support the well-being of the population, and the influence of Keynesian economics. On the one hand, there were strong social determinants of health voices that saw a natural alliance between public health and welfare services as partners in population well-being. On the other hand, the downstream and patronizing orientation seen in earlier public health activities persisted. An example is an article by Severs et al. (1961) on infantile scurvy, where social and economic contributing factors were recognized but the recommended solutions were around education of mothers, consistent advice by health professionals, and fortification of milk with vitamin C.

A second observation was the apparent escalation of economic rationalization, which now extended to include elements of accountability, evaluation, and efficiency. This emerged prominently in discussions about hospitals, which—likely spurred by the 1961 federal Hospital Insurance and Diagnostic Services Act^5^—had a much greater presence in Volume 52 than we had seen previously. Articles frequently expressed a particular logic that privileged questions about the value of a range of health services to be answered through formalized processes of evaluation, in which cost efficiencies figured prominently. A paper by ***Schwenger*** (1961), for example, described a program evaluation of local health programs, situated in a context of apparent anxiety about public health justifying the federal health grants to the provinces. The implicit, and often explicit, question underpinning the work was whether the public were getting their money’s worth, which could be answered via formal evaluation with an emphasis on systematic procedures, including epidemiologic objectivity and precision.

These trends struck us as doubly problematic because of (a) enhanced economic rationalization as the foundation for public health concerns and activities, and (b) the increasing focus on hospitals, a clear example of a downstream creep in public health. Although we are very familiar with these trends today, it was surprising to us to see this kind of orientation as early as 1961. In the light of these trends, it is perhaps not surprising that Volume 52 also contained some pieces reflecting on the scope and contours of public health vis-à-vis other fields, including clinical medicine (e.g., Greenhill, 1961).

## 1995, Volume 86: Upstream health determinants and uncritical epidemiology: Uneasy bedfellows

Volume 86 strongly resembled the journal we know today in appearance and format, with six issues per year featuring predominantly original empirical, mostly quantitative, research, and literature reviews presented in a structured format with a bilingual abstract. Research papers frequently had multiple authors, signalling the newer presence of research *teams*. In line with a clear focus on research (more so than on practice/policy), the journal’s employment advertisements in 1995 were mostly for academic positions.

In the wake of the Ottawa Charter for Health Promotion (WHO, 1986) and other significant publications (e.g., Evans et al., 1994), health promotion and population health were prominent in Volume 86. Epidemiologic papers were frequently framed as informing health promotion programming, and a preoccupation with the emerging field was evident from studies assessing health promotion training programs—posing questions such as what skills are needed in the emerging field and who gets to define them (e.g., Allison et al., 1995; Green, 1995).

Volume 86 was published during the economic recession of the 1990s with its corresponding neoliberal discourse and policy emphasis on debt and deficit reduction and cuts to public services. In that context, there was some critically oriented engagement with economic policy. This includes an editorial by Reutter (1995) which argued that the public health community has a responsibility to advocate for changes to economic policy to reduce poverty and its health implications, a piece by Frank (1995) which was unambiguous in its argument around the political economic commitment that is required of a genuine embrace of population health, and an editorial by Draper (1995) which noted the contradiction between public health’s emphasis on socio-economic determinants of health and its complicity around dominant economic policy that dismantles the welfare state and permits the growth of international power. A piece by ***Labonté*** (1995) stood out; in his discussion of tensions between and within health promotion and population health, Labonté’s cogent critique of economic liberalism from a health equity perspective was one that we felt could have been written today.

Unfortunately, these critical perspectives were limited to conceptual pieces. In terms of empirical research, Volume 86 contained an abundance of epidemiologic articles framed in a downstream and decontextualized manner. For example, one paper addressed the important issue of socio-economic patterning of breastfeeding, but stopped short of identifying any upstream causes, or implications, of the findings, landing instead on the typical public health recommendation to educate target groups (Nolan & Goel, 1995). Another paper, on air quality in urban Toronto (Campbell et al., 1995), concluded with concern about air quality worsening as urban density increases, but stopped short of drawing any connections to the organization of the economy that permitted the production of air toxins in the first place.

## 2013, Volume 104: Methods fixation and economic policy at the margins

Our final volume for analysis falls into what Osberg (2021) calls “zombie neoliberalism”. Considering the need and potential for a significant political economic shift, one might expect to see meaningful public health engagement with matters of the economy in this context. What we observed, however, was largely the opposite, with even less engagement than in 1995. Indeed, a key observation for Volume 104 was that the journal’s zeitgeist was not very apparent at all.

While Volume 104 evinced a massive expansion of topics, including some that are at best on the periphery of public health, we also observed an amplification of a trend seen in 1995, namely, a proliferation of uncritical, incremental, quantitative studies. In 2013, these often took the form of a hyper-specified focus on comparing clinical or behavioural variables among groups of people. This volume thus struck us as illustrative of a technical retreat, meaning a fixation on data and epidemiologic methods rather than on political economic circumstances. While we noted a growing emphasis on the health of Indigenous Peoples in Volume 104, those articles likewise tended to be narrow, decontextualized epidemiologic comparisons. By 2013, the journal consisted overwhelmingly of empirical papers, and implications—when they were drawn—were more often for research than for practice or policy. When practice/policy implications were drawn, they were frequently vague (for example, that a particular risk factor “should be considered” in preventive interventions, or that a certain population group “requires increased support” to adopt better behaviour).

In 2013, we observed even deeper retreat into the, by this time, long-standing tendency to justify a health problem’s importance using its monetary cost, now usually with a precise dollar amount attached. There was also a strong presence of cost-effectiveness claims, and newer approaches like data linkage were touted for their ability to inform cost-effective health care in a context of economic constraints—thus illustrating acceptance of the neoliberal status quo of austerity. While a few pieces engaged with the economy in the form of private commercial enterprises (e.g., the food industry), with few exceptions (e.g., Millar, 2013), these papers tended to focus on policy at the margins (e.g., food packaging labels) and/or to engage with industry in a way that tried to accommodate its priorities (e.g., to seek “win–win” arrangements for business and for health). This struck us as a significant contrast to the strongly anti-corporate stance in earlier scholarship around tobacco.

Some authors in Volume 104 engaged thoughtfully with matters of the economy. Gore and Kothari (2013), for example, reviewed healthy living initiatives in British Columbia and Ontario with the aim of clarifying and encouraging structural approaches. However, although it mentioned “economic” several times, the meaning of the word was unspecified. Cohen et al. (2013) aimed to determine how to build more public health capacity around upstream social determinants of health. Despite its equity focus, that paper was largely silent on economic issues. A rare example of research engaging critically with matters of the economy was a study by ***Collins and Hayes*** (2013), which was situated in the Healthy Cities movement. Through interviews with municipal authorities, it illuminated that, although municipal governments did have program and policy levers for acting on social determinants of health, a (problematic) behaviourist bent emphasized parks and recreation as the main mechanisms to address health inequities.

Although the notion of health equity figured prominently in the journal in 2013, thoughtful engagement with economics and economic policy was rare. The far more dominant tendency was for health inequity to be framed in a myopic, technical sense, focusing on measurement, with at most non-committal language around upstream implications. Moreover, public health’s long-standing tendency to moralize persisted, where people were constructed as deviant and deficient for any number of reasons—too much drinking or smoking, having unsafe sex, or being too fat. Overall, this volume left us feeling that the CJPH community was not rising to the challenge of contemporary health problems and health inequities, and wondering what it would take for this to change.

## Conclusions

There is growing awareness and scholarship around the immense consequences for health of our political economic system, and the urgent need for a new one. We reviewed and analyzed select historical volumes of the CJPH to explore how past engagement in economic issues and economic policy, including government policy and its broader political economic context, by the Canadian public health community might instruct efforts to encourage critical engagement in economic policy by our field.

Our analysis suggests that, at least in terms of the historical record, public health researchers in Canada are ill equipped to seriously engage with economic policy issues from a robust public health perspective. We found only a slim historical foundation for public health engagement with the economy. Across all time periods, and notwithstanding changes in the journal’s authorship and styles of written communication, connections between the economy and health were rarely made. Explicit interventions in or commentary on economic policy and its health effects were even rarer. Moreover, we observed a strong and apparently subconscious allegiance to dominant economic paradigms, despite their incompatibility with root causes of health inequities. This allegiance manifested as a tendency, dating to the earliest issues, to rationalize health problems and interventions based on their monetary cost rather than on the inherent value of human life. Finally, even though socio-economic inequalities in health are a long-standing preoccupation of CJPH authors, those inequalities are consistently and curiously divorced from their roots in economic systems, with authors instead defaulting to downstream and frequently patronizing and exclusionary solutions. We were disappointed to see that, when economic circumstances and policy environment were especially problematic (e.g., during the Great Depression, and the Neoliberal era), public health seemed to retreat even further into economic rationalization, downstream solutions, and fixation on methods, rather than prompting coordinated efforts for change.

To be sure, we identified several important examples of thoughtful and critical engagement by CJPH authors with matters of the economy. In the mid-1990s, the appearance of a discourse on upstream socio-economic determinants of health helped leverage public health critiques of liberal economic policy. That impulse needs to be strengthened not only with further conceptual critiques of economic liberalism in the pages of CJPH, but with empirical research exploring the relationship between economic policy decisions and population-level health outcomes. Overall, we argue—as others have done before us—that the continued dominance of implicit and explicit economic liberalism and residualism in the field, which is intertwined with its medical origins and manifests in, for example, reductive and under-theorized engagement with the relationship between income and health (Raphael et al., 2006), is obstructing our community’s ability to work coherently towards our own stated goals of population well-being and health equity, which are fundamentally rooted in our economic, and other, systems. As noted elsewhere in this journal, part of the challenge is to get our own house in order (McLaren & Hancock, 2019). As one step towards that goal, we enthusiastically recommend the highly informative exercise of reading the journal’s history.

Lindsay McLaren, Senior Editor, CJPH

Eric Mykhalovskiy, Associate Editor, CJPH

## Commentaire

### Introduction

Il est bien établi que le bien-être des populations et l’équité en santé sont influencés de façon significative par les déterminants sociaux de la santé, y compris les politiques publiques en amont et leurs fondements idéologiques (CSDH, 2008; Raphael et al., 2020). Les politiques économiques mises de l’avant par les gouvernements contribuent beaucoup à structurer les déterminants de la santé et leur répartition. Citons par exemple les dépenses pour les services publics et les mesures de soutien du revenu; les politiques fiscales qui structurent la répartition des revenus et de la richesse; la libéralisation des activités financières et des échanges commerciaux, qui affecte la répartition mondiale des revenus et de la richesse et bouleverse les marchés du travail en créant des emplois pour certains et en précarisant ceux des autres; et la réglementation des industries extractives et des écosystèmes et ses répercussions sur le climat et d’autres aspects de la santé environnementale (NCCDH, 2022; CPHA, 2015).

Malgré l’importance de la politique économique pour la santé, l’implication des communautés de la santé publique à cet égard a tendance à être limitée, se concentrant par exemple sur le rendement du capital investi dans les interventions de santé publique (Masters et al., 2017) ou sur la part des dépenses du secteur de la santé consacrée aux activités de santé publique (Fiset-Laniel et al., 2020; McLaren & Dutton, 2020). Une plus grande implication dans les déterminants en amont, tels qu’incarnés dans la politique économique, serait pourtant une bonne façon de renforcer les conditions du bien-être des populations et de l’équité en santé, qui sont les objectifs déclarés de la santé publique (CPHA, 2017).

Bien qu’elle constitue un bon levier pour renforcer le bien-être des populations et l’équité en santé, la politique économique ne sert clairement pas à cette fin au Canada, ni à vrai dire dans la plupart des pays du monde. Les effets considérables et très inéquitables des crises financières mondiales à répétition, de l’escalade des changements climatiques et de la dégradation de l’environnement, et ceux de la pandémie de COVID-19, ne laissent aucun doute quant à l’incompatibilité entre le capitalisme néolibéral (le système économique dominant dans le monde depuis le début des années 1980) et les déterminants sociaux et écologiques de l’équité en santé (Bump et al., 2021; Ruckert & Labonté, 2014). Le monde a désespérément besoin d’un nouveau paradigme politico-économique. Comment faire pour inciter la communauté de la santé publique canadienne à se mobiliser utilement en faveur d’un système économique qui favorise le bien-être des populations et l’équité en santé, ce qui inclut les politiques économiques mises de l’avant par les gouvernements et le paradigme politico-économique dans lequel elles s’inscrivent? Pour répondre à cette question, tournons-nous vers un examen historique de l’implication de la santé publique dans la politique économique. En nous concentrant spécifiquement sur la *Revue canadienne de santé publique* (RCSP), qui s’intéresse depuis longtemps à la prévention et au bien-être,^6^ nous avons étudié les bases historiques de l’implication actuelle de la santé publique dans la politique économique, puis cherché à tirer des leçons des démarches adoptées par les générations passées d’acteurs de la santé publique pour repérer les liens entre l’économie et la santé et agir en conséquence.

Nous avons divisé l’histoire de la Revue (depuis 1910)^7^ en six périodes correspondant à des circonstances ou à des événements économiques phares : 1910 à 1928 (du premier numéro de la Revue aux suites de la Première Guerre mondiale); 1929 à 1938 (du krach boursier à la Grande Dépression); 1939 à 1945 (la Deuxième Guerre mondiale, du point de vue des préoccupations économiques); 1946 à 1979 (la période keynésienne); 1980 à 2007 (les débuts du néolibéralisme); et 2008 à 2021, période qu’Osberg (2021) qualifie de « néolibéralisme zombie », car malgré l’éclatement de la crédibilité du néolibéralisme, aucun paradigme de rechange n’a encore émergé. Pour chacune de ces périodes, nous avons sélectionné au hasard un volume (une année complète), que nous avons lu en entier en nous attachant : au contexte et aux grands événements historiques; aux sujets abordés; au type d’auteurs et à leur diversité; à l’analyse des questions économiques; et à l’analyse des interventions économiques dans l’intérêt de la santé (au sens large). Nous nous sommes rencontrés pour comparer et réviser nos notes d’analyse sur chaque volume choisi. Nous avons sélectionné d’après leur valeur heuristique certains articles,^8^ indiqués ci-après en ***caractères gras italiques***, que nous présentons intégralement dans cette section spéciale de la Revue.

## 1917 (volume 8) : Hygiène publique, économie, eugénisme

Outre le fait qu’elle porte un autre nom, la RCSP de 1917 ne ressemble en rien à la Revue d’aujourd’hui. On y trouve très peu d’études empiriques; elle est constituée dans une large mesure de comptes rendus de congrès et de réunions, et de transcriptions d’allocutions et de discours locaux. Ces allocutions sont prononcées, presque sans exception, par des médecins de sexe masculin exerçant des fonctions d’agents d’hygiène publique au Canada ou ailleurs (d’habitude aux États-Unis ou en Grande-Bretagne). Les annonces publicitaires de la Revue donnent l’impression que l’un de ses premiers rôles est de créer un marché pour certains produits et services : les voyages internationaux, les instruments médicaux et les produits de nettoyage. Pour inscrire les préoccupations actuelles dans une perspective historique, disons que le volume 8 déplore la mésinformation (les charlatans), le non-respect des règles d’hygiène publique et le manque de compréhension et d’appréciation pour ce domaine au sein de la population.

Dans le contexte de la Première Guerre mondiale, certains auteurs reconnaissent que les facteurs économiques sont la cause profonde des maladies, mais non sans exprimer des relents de sexisme, de racisme et de classisme. Graham (1917), par exemple, parle des conséquences pour la santé mentale « du bilan catastrophique des combats et de la pauvreté abjecte » qui poussent naturellement les travailleurs à chercher un soulagement dans l’alcool, entre autres. La guerre, soutient Graham, donne aux gens le sentiment du devoir et devrait de ce fait réduire la maladie mentale, surtout chez les femmes de la classe moyenne qui découvrent que la vie est plus que « les tièdes ragots d’un club de couture paroissial ». On trouve aussi dans le volume 8 les premiers signes d’une rationalisation économique : seul le coût monétaire d’un problème de santé justifie l’attention qu’on y accorde. Entre autres exemples en cette période de guerre, citons les inquiétudes soulevées par le coût des maladies vénériennes, dont les traitements médicaux sont chers (Hastings, 1917), et par le recrutement de soldats infectés par la tuberculose (Hattie, 1917) (la pandémie d’influenza de 1918‒1919 et les préoccupations économiques considérables qu’elle va probablement engendrer dans la communauté de la santé publique sont encore loin). On a aussi recours à un raisonnement économique pour mobiliser l’opinion en faveur de l’eugénisme : Knopf (1917), par exemple, fait valoir que la contraception réduit le nombre d’enfants physiquement, mentalement et moralement inaptes, qu’il caractérise comme coûtant cher à soigner et sapant la vigueur de la population.^9^

Nous relevons dans le volume 8 quelques exemples de l’implication de la communauté de la santé publique canadienne dans la politique économique et les mécanismes étatiques pour aborder les problèmes ancrés dans les conditions économiques. Un article de McCullough (1917), par exemple, mentionne que les propriétaires d’usines de munitions sont autorisés à prolonger les heures de travail pour soutenir l’effort de guerre. Bien que l’article ne mentionne pas les mots « économie » ni « économique », il recommande une réduction de la journée de travail, notamment pour des raisons de santé. Dans un article faisant l’apologie des familles nombreuses, Weston (1917) préconise une politique économique qui compense le coût financier d’avoir des enfants. Il demande spécifiquement l’imposition d’une taxe à chaque ménage en vue de redistribuer les fonds aux familles nombreuses. Notre troisième exemple est un article sur le logement rédigé par ***Cartlidge*** (1917), l’inspecteur-hygiéniste en chef de Moose Jaw, en Saskatchewan. Anticipant une « vague d’immigration » après la guerre, Cartlidge prône la réglementation des constructeurs sans scrupules « dont le seul but est de construire à bas prix » pour ensuite louer cher. Nous sommes frappés par la pertinence toujours actuelle de cet article de 1917, qui fait le lien entre l’hygiène publique et la dynamique de pouvoir enchâssée dans le marché de l’habitation et le développement urbain.

## 1933 (volume 24) : Repli sur soi en temps de crise économique

En 1933, dans le contexte de la Grande Dépression, la communauté de la RCSP réagit clairement en se repliant sur soi et en baissant la tête. Au lieu d’aborder directement les conséquences probablement graves des conditions économiques sur la santé, les auteurs semblent considérer la dépression comme un problème quasi insurmontable. Ils ont d’ailleurs plus tendance à en déplorer les effets néfastes sur le nombre de membres de l’ACSP et de commanditaires de la Revue que sur la santé du public.

Dans le prolongement de ce repli sur soi, le volume 24 exprime une orientation biomédicale marquée. De nombreux articles portent sur les aspects médicaux ou cliniques de maladies particulières (tuberculose, variole, rougeole) en faisant abstraction de leur contexte. Dans son analyse de la diarrhée et de l’entérite, par exemple, McKinnon (1933) parle des autres diagnostics médicaux possibles et des mouches comme étant sources de bactérioses, mais il passe sous silence les conditions sociales et économiques qui rendent les gens plus exposés ou plus vulnérables aux vecteurs de maladies. Les articles de ce genre, axés sur la maladie, prennent souvent la forme de rapports épidémiologiques; à partir de cette époque en effet, la Revue se concentre nettement sur les données et les chiffres, comme en témoigne une rubrique intitulée « Épidémiologie et statistiques de l’état civil » (*Epidemiology and Vital Statistics*).

Les articles sur les aliments et la nutrition illustrent bien la nature de l’implication de la RCSP dans les questions économiques en 1933. Le numéro 5 contient plusieurs articles sur les moyens possibles d’intégrer des éléments de nutrition dans les programmes d’aide de l’État. Un article de ***McHenry*** (1933) est représentatif de l’orientation de santé publique dominante à l’époque : aider les gens à s’adapter aux conditions économiques (implicitement fixes) en les amenant à prendre de meilleures décisions (plus nutritives, plus prudentes) concernant le régime alimentaire familial. De même, Bell (1933) veut aider les gens à faire face à la calamité économique et sociale de la dépression en intégrant la science émergente de la nutrition dans les prudents conseils de santé publique sur la nourriture. Goodeve (1933) décrit le travail des responsables des secours alimentaires à Montréal, qui consiste à apprendre aux mères à faire de meilleurs choix, et déplore « ces infortunées qui n’ont jamais appris à dépenser au mieux leur argent ». Les initiatives de ce genre, qui s’adressent presque toujours aux mères, illustrent les racines historiques des préjugés sexistes, hétéronormatifs, classistes (favorisant la classe moyenne) et racistes (favorisant la race blanche) du « projet moral » de la santé publique d’aujourd’hui.

On trouve peut-être ce qui se rapproche le plus d’une analyse critique de la politique économique en 1933 dans les écrits sur les maladies professionnelles et la santé au travail. Un éditorial dans le numéro 4, par exemple, déplore le fait que la protection contre les dangers en milieu de travail est essentiellement laissée aux entreprises et aux usines, qui s’en acquittent comme bon leur semble, au gré de leurs intérêts commerciaux, tandis que « les lois et les inspections viennent loin derrière » (Cunningham, 1933). Les commentaires de la sorte font allusion au besoin de fixer des limites à l’activité économique pour protéger la main-d’œuvre, mais ne vont pas jusqu’à dire que cette dynamique politico-économique devrait préoccuper davantage dans un domaine qui se soucie de la santé du public. Dans l’ensemble, la nature de l’implication de la RCSP dans la politique économique en 1933 nous semble en fait représenter un recul par rapport à 1917.

## 1941 (volume 32) : Guerre, économie, santé de la nation

Publié durant la Deuxième Guerre mondiale, le volume 32 contient des indices que les auteurs commencent à faire le lien entre la guerre, les questions économiques et la santé publique. En général, ces liens révèlent la même rationalisation économique que par le passé : la santé de la nation est décrite comme un bien économique (autrement dit, nécessaire pour combler les besoins industriels et militaires de l’effort de guerre) auquel la santé publique peut contribuer au moyen d’interventions efficaces sur le plan économique et en faisant appel à la science, laquelle est nettement présente dans la Revue à partir de 1941.

Ce discours prend plusieurs tournures. L’une d’elles, qui reprend un thème de 1933, est la publication d’articles sur l’état nutritionnel de la population. Les iniquités sur le plan de la nutrition, tributaires des conditions économiques, sont manifestes et reconnues comme étant causées par l’insuffisance des revenus, mais les auteurs de la RCSP continuent de préconiser la pratique condescendante qui consiste à aider des gens à faire des choix plus intelligents et plus économes dans leurs achats et leur cuisine (p. ex. Hunter & Pett, 1941). En 1941, on insiste beaucoup sur le lait, promu comme un « super-aliment » avec une ferveur proche de la propagande. Des articles sur divers aspects du lait (sa pasteurisation, son étiquetage, sa distribution, sa consommation) illustrent l’acceptation par la santé publique du statu quo économique. Par exemple, un article fait l’éloge de la pasteurisation obligatoire en Ontario en disant qu’elle ne nuit pas à l’industrie laitière (éditorial, 1941, numéro 4). Emblématique de l’orientation de la santé publique, un article de ***Kitching*** (1941) présente les résultats d’un sondage sur la consommation de lait des « groupes familiaux » de Vancouver. Constatant sans surprise que cette consommation est faible et inégale à l’échelle de la population, Kitching recommande de faire de la sensibilisation, notamment auprès des « femmes au foyer » dans les familles à faible revenu, pour qu’elles « se concentrent sur les priorités », comme le dit l’éditorial de ce numéro.

Les rapprochements entre la guerre, l’économie et la santé publique se manifestent d’une deuxième façon : par la publication d’articles sur la santé et la sécurité de la main-d’œuvre. La Revue comporte même une rubrique intitulée « Hygiène industrielle » (*Industrial Hygiene*). La santé des travailleurs est souvent exprimée en termes économiques (p. ex. les coûts monétaires des journées de travail perdues) pour en justifier l’importance. Reprenant une observation datant de 1917, les auteurs du volume 32 tirent des conclusions bien pensées entre les conditions de travail et la santé. Selon un résumé publié à la rubrique « Hygiène industrielle » du numéro 2, par exemple, le taux de suicide élevé chez les cheminots est lié aux « conditions économiques précaires », ce qui fait penser à des articles plus récents sur l’économie politique (p. ex. Stuckler & Basu, 2013). Dans certains cas, ces liens se traduisent par une attention à la politique économique. Un résumé publié à la rubrique « Hygiène industrielle » du numéro 10 recommande par exemple au ministère du Travail – vu les heures supplémentaires et la fatigue excessive de la main-d’œuvre – de réduire la durée de la semaine de travail.

Troisièmement, l’imbrication de la guerre, de l’économie et de la santé en 1941 incite certains auteurs de la RCSP à préconiser une redistribution à grande échelle des ressources pour améliorer la santé, surtout celle des personnes sous le seuil de la pauvreté. McCann (1941), par exemple, réclame d’importants ajouts à l’État providence, sachant « que la maladie et la pauvreté vont toujours ensemble, mais que la pauvreté est à mon avis davantage la cause de la maladie que son résultat (…). La solution (…) est d’ordre social et économique. » Néanmoins, le projet moral raciste et classiste de la santé publique continue de poindre son vilain nez, comme dans la recommandation de Dolman (1941), selon qui les prestations fiscales devraient servir à encourager les familles de la classe moyenne à faire plus d’enfants pour sauver la nation.

Bien qu’ils ne datent pas de 1941, les dons de bienfaisance et le financement de société sont très présents dans le volume 32. Cette présence, qui n’a rien d’anodin dans un domaine qui se préoccupe de la santé du public, inclut le soutien financier de la Fondation Rockefeller, de la Canadian Life Insurance Officers Association, de La Métropolitaine, Compagnie d’Assurance-Vie et de la Fondation Kellogg aux activités de l’ACSP (numéro 7).

## 1961 (volume 52) : État providence, efficience, responsabilité

Le contexte de la guerre froide, dans le volume 52, se manifeste surtout par l’attention accordée aux « nouvelles » questions de santé publique : la guerre nucléaire, les armes biologiques et chimiques et les retombées radioactives. Malgré leurs liens clairs avec l’économie politique mondiale, notamment la concurrence entre le modèle de marché planifié par l’État de l’URSS (Union des républiques socialistes soviétique) et le modèle libéral dont les États-Unis sont le fer de lance, ces questions sont généralement abordées dans une optique clinique ou biomédicale. Les maladies chroniques commencent à préoccuper la communauté de la RCSP en 1961, et leur émergence s’accompagne d’un intérêt, quoique limité, pour les affaires économiques; on déplore par exemple les « forces économiques » (non précisées, mais qui désignent probablement le pouvoir économique et politique de l’industrie du tabac) qui contrecarrent les efforts de prévention du cancer du poumon. La Revue continue d’inclure des transcriptions de discours prononcés lors de congrès, mais le volume 52 contient une proportion croissante de documents de recherche (dont quelques articles en français). Cela pourrait s’expliquer par la création, en 1960, du Conseil de recherches médicales du Canada, précurseur des Instituts de recherche en santé du Canada (numéro 2).

En ce qui a trait au rôle de la santé publique dans les politiques économiques, nous observons deux choses dans le volume 52. La première est la relation apparemment tendue entre la santé publique et les services sociaux. Elle s’inscrit dans le contexte de l’émergence de l’État providence, qui reflète à son tour les épreuves sociales et économiques de la Grande Dépression, le besoin clair de systèmes étatiques pour favoriser le bien-être de la population, et l’influence de la pensée économique keynésienne. D’une part, d’ardents défenseurs des déterminants sociaux de la santé voient une alliance naturelle entre la santé publique et les services sociaux, pour le bien-être de la population. D’autre part, l’approche condescendante, du sommet vers la base, que nous avons vue dans les activités de santé publique antérieures ne disparaît pas. On en trouve un exemple dans un article de Severs et al. (1961) sur le scorbut infantile : l’auteur reconnaît les facteurs sociaux et économiques qui contribuent à cette maladie, mais les solutions qu’il recommande sont d’éduquer les mères, d’enjoindre les professionnels de la santé à tous prodiguer les mêmes conseils et d’enrichir le lait avec de la vitamine C.

Nous observons également une intensification apparente de la rationalisation économique, qui comprend désormais des éléments de responsabilité, d’évaluation et d’efficience. Cela se manifeste clairement lorsqu’il est question des hôpitaux, lesquels (probablement sous l’impulsion de la *Loi fédérale sur l’assurance-hospitalisation et les services diagnostiques* de 1961^10^) sont beaucoup plus présents dans le volume 52 qu’ils ne l’étaient auparavant. Les articles expriment souvent une logique particulière qui privilégie les questions sur l’utilité d’un éventail de services de santé, questions auxquelles il faut répondre selon un processus d’évaluation formalisé, très axé sur le rapport coût-efficacité. Un article de ***Schwenger*** (1961), par exemple, porte sur l’évaluation de programmes de santé locaux dans le contexte d’un souci apparent que la santé publique justifie les allocations pour la santé consenties par le gouvernement fédéral aux provinces. La question qui sous-tend ce travail implicitement, et souvent explicitement, est de savoir si le public en a pour son argent, ce qui peut être déterminé au moyen d’une évaluation formelle menée selon des méthodes systématiques, dont l’objectivité et la précision épidémiologiques.

Ces tendances nous semblent doublement problématiques en raison : a) de la rationalisation économique plus prononcée qui forme la trame des questions et des activités de santé publique; et b) de l’intérêt croissant pour les hôpitaux, un exemple clair d’un glissement vers l’aval en santé publique. Ces tendances sont monnaie courante aujourd’hui, mais nous avons été étonnés de les repérer dès 1961. Il est peut-être donc logique que le volume 52 renferme aussi des articles qui s’interrogent sur la portée et les contours de la santé publique par rapport à d’autres domaines, dont la médecine clinique (p. ex. Greenhill, 1961).

## 1995 (volume 86) : Un mauvais ménage entre les déterminants de la santé en amont et l’épidémiologie dépourvue d’esprit critique

Le volume 86 ressemble beaucoup à la Revue que nous connaissons aujourd’hui, dans son apparence comme dans son format, avec six numéros par année qui contiennent principalement des études empiriques originales, quantitatives pour la plupart, et des revues de la littérature spécialisée présentées sous une forme structurée avec un résumé bilingue. Les documents de recherche ont souvent plusieurs auteurs, signe de la nouvelle présence *d’équipes* de recherche. Clairement axée sur la recherche (plus que sur les pratiques ou les politiques), la Revue publie surtout des offres d’emploi pour des postes universitaires en 1995.

Dans la foulée de la Charte d’Ottawa pour la promotion de la santé (WHO, 1986) et d’autres publications phares (p. ex. Evans et al., 1994), la promotion de la santé et la santé des populations sont bien en vue dans le volume 86. Les articles de nature épidémiologique sont souvent présentés comme pouvant éclairer les programmes de promotion de la santé, et une préoccupation pour ce domaine émergent ressort clairement des études d’évaluation des programmes de formation en promotion de la santé, où l’on demande quelles sont les compétences nécessaires dans ce domaine émergent et à qui revient la tâche de les définir (p. ex. Allison et al., 1995; Green, 1995).

Le volume 86 est publié durant la récession économique des années 1990, avec le discours néolibéral de l’époque et ses prises de position sur la réduction de la dette et du déficit et sur les compressions dans les services publics. Dans un tel contexte, la politique économique est parfois critiquée. Un éditorial de Reutter (1995) fait valoir que la communauté de la santé publique a l’obligation de préconiser des changements dans la politique économique pour réduire la pauvreté et ses conséquences pour la santé; un article de Frank (1995) déclare sans équivoque que pour épouser la cause de la santé des populations, il faut un engagement politico-économique; et un éditorial de Draper (1995) souligne la contradiction entre l’importance que la santé publique accorde aux déterminants socioéconomiques de la santé et sa complicité envers la politique économique dominante qui démantèle l’État providence et permet la croissance du pouvoir dans le monde. Un article de ***Labonté*** (1995) se démarque du reste; dans son analyse des tensions entre la promotion de la santé et la santé des populations et à l’intérieur de ces deux domaines, Labonté formule une critique convaincante du libéralisme économique dans une optique d’équité en santé toujours actuelle selon nous.

Malheureusement, ces perspectives critiques sont limitées aux articles théoriques. En matière de recherche empirique, le volume 86 contient une foule d’articles épidémiologiques présentés selon une démarche d’aval décontextualisée. À titre d’exemple, un article aborde l’importante question de la structuration socioéconomique de l’allaitement maternel, mais ne va pas jusqu’à nommer les causes en amont, ni les conséquences, de ses constats, se contentant plutôt d’émettre la recommandation de santé publique type : éduquer les groupes cibles (Nolan & Goel, 1995). Un autre article, sur la qualité de l’air dans la région urbaine de Toronto (Campbell et al., 1995), conclut que l’aggravation de la qualité de l’air avec la densification urbaine est préoccupante, mais ne va pas jusqu’à faire un rapprochement avec l’organisation de l’économie, qui permet la production de toxines atmosphériques en premier lieu.

## 2013 (volume 104) : Fixation sur les méthodes, politique économique marginale

Le dernier volume que nous avons analysé s’inscrit dans ce qu’Osberg (2021) appelle le « néolibéralisme zombie ». Étant donné le besoin (et le potentiel) d’un changement politico-économique de taille, on aurait pu s’attendre à voir la santé publique jouer un grand rôle dans les questions économiques. Dans une large mesure, nous observons toutefois le contraire : ce rôle est encore moins important qu’il ne l’était en 1995. En fait, l’une de nos principales observations à l’égard du volume 104 est que l’esprit du temps a peine à effleurer dans la Revue.

Le volume 104 révèle un foisonnement de nouveaux sujets, dont certains se situent, au mieux, en périphérie de la santé publique, mais nous observons aussi l’amplification d’une tendance notée en 1995, à savoir la prolifération d’études quantitatives, incrémentielles et dépourvues d'esprit critique. En 2013, ces études prennent souvent la forme d’une hyperfocalisation sur la comparaison des variables cliniques ou comportementales de différents groupes. Ce volume nous paraît donc illustrer un repli technique : une fixation sur les données et les méthodes épidémiologiques plutôt que sur la situation politico-économique. Nous remarquons bien un intérêt croissant pour la santé des peuples autochtones dans le volume 104, mais les articles en question tendent eux aussi à être des comparaisons épidémiologiques étroites et décontextualisées. En 2013, la Revue se compose très majoritairement d’articles empiriques, et leurs implications – lorsqu’elles sont mentionnées – concernent plus souvent la recherche que les pratiques ou les politiques. Quand il y a des implications pour les pratiques ou les politiques, elles sont souvent vagues (par exemple, qu’un facteur de risque particulier « devrait être pris en compte » dans les mesures de prévention ou qu’une certaine population « a besoin de plus de soutien » pour adopter un meilleur comportement).

Nous observons en 2013 un repli encore plus marqué vers la tendance déjà ancienne à justifier l’importance d’un problème de santé en citant ses coûts monétaires, à la différence qu’on y rattache maintenant très souvent un montant précis. Les allégations d’efficacité par rapport au coût sont aussi très présentes : de nouvelles méthodes, comme le couplage de données, sont vantées pour leur possibilité d’éclairer l’efficacité des soins de santé par rapport à leur coût dans un contexte de contraintes économiques, ce qui dénote l’acceptation du statu quo néolibéral de l’austérité. Un petit nombre d’articles parlent d’économie sous le visage de l’entreprise privée (p. ex. l’industrie alimentaire), mais à quelques exceptions près (Millar, 2013), ils ont tendance à porter sur des politiques marginales (comme l’étiquetage des aliments) ou à aborder l’industrie en essayant de tenir compte de ses priorités (p. ex. en cherchant des solutions « gagnant-gagnant » pour les entreprises et pour la santé). Cela nous semble très différent de la position résolument anti-entreprises des articles antérieurs sur le tabagisme.

Certains auteurs du volume 104 réfléchissent attentivement aux questions économiques. Gore et Kothari (2013), par exemple, passent en revue des initiatives sur les modes de vie sains menées en Colombie-Britannique et en Ontario afin de clarifier et d’encourager les approches structurelles. Le mot « économique » est cité à plusieurs reprises sans toutefois être défini. Cohen et collègues (2013) cherchent à trouver des moyens de renforcer les capacités de la santé publique à l’égard des déterminants sociaux de la santé en amont. Bien qu’il porte sur l’équité, leur article reste généralement muet sur les questions économiques. Une étude de ***Collins et Hayes*** (2013) qui s’inscrit dans le mouvement des Villes-santé est l’un des rares exemples d’un regard critique sur les questions économiques. Au moyen d’entretiens avec des conseillers municipaux, Collins et Hayes montrent que même si les administrations municipales disposent de certains leviers (des programmes, des politiques) pour agir sur les déterminants sociaux de la santé, une tendance behavioriste douteuse privilégie les parcs et les loisirs comme principaux moyens de résoudre les iniquités en santé.

La notion d’équité en santé a beau occuper une place prépondérante dans la Revue en 2013, il est rare d’y trouver une réflexion attentive sur l’économie et la politique économique. La tendance de loin la plus présente consiste à aborder les iniquités en santé selon une vision technique à courte vue, en se contentant de les mesurer et en utilisant un langage évasif pour en décrire les origines. De plus, la tendance moralisatrice bien établie en santé publique est toujours présente; les gens sont dépeints comme étant déviants et déficients pour toutes sortes de raisons : ils boivent et fument trop, ils ont des rapports non protégés, ils sont trop gros. Dans l’ensemble, ce volume donne l’impression que la communauté de la RCSP ne se montre pas à la hauteur devant les problèmes de santé et les iniquités en santé de l’époque, et on se demande ce qu’il faudrait pour que cela change.

## Conclusions

Les conséquences énormes du système politico-économique sur la santé – et la nécessité urgente d’un nouveau système – sont de plus en plus reconnues et étudiées. Nous avons sélectionné et analysé d’anciens volumes de la RCSP pour voir comment l’implication passée de la communauté de la santé publique canadienne dans la politique et les enjeux économiques, y compris les politiques gouvernementales et le contexte politico-économique dans lequel elles se situent, pourrait éclairer des démarches incitant notre domaine à aborder la politique économique d’un œil critique.

Notre analyse indique que sur le plan historique à tout le moins, les chercheurs en santé publique au Canada sont mal équipés pour aborder sérieusement la politique économique dans une perspective de santé publique. Les bases historiques de l’implication de la santé publique dans l’économie sont ténues. À toutes les époques, peu importe les changements dans les auteurs et les styles de rédaction de la Revue, on fait rarement un rapprochement entre l’économie et la santé. Les interventions ou les commentaires visant explicitement les effets de la politique économique sur la santé sont encore plus rares. Par ailleurs, nous observons une allégeance solide et apparemment inconsciente envers les paradigmes économiques dominants, malgré leur incompatibilité avec les causes profondes des iniquités en santé. Cette allégeance se manifeste dans une tendance, qui remonte aux tout premiers numéros, à rationaliser les problèmes de santé et les interventions connexes en fonction de leurs coûts monétaires et non de la valeur inhérente de la vie humaine. Enfin, même si elles préoccupent depuis longtemps les auteurs de la RCSP, les inégalités socioéconomiques en santé sont constamment et curieusement divorcées de leurs racines dans les systèmes économiques; au lieu de cela, les auteurs se tournent par défaut vers des solutions d’aval souvent condescendantes et exclusives. Nous sommes déçus de constater que lorsque les conditions économiques et l’environnement politique sont particulièrement difficiles (comme pendant la Grande Dépression et à l’ère du néolibéralisme), la santé publique semble se replier encore davantage sur la rationalisation économique et les solutions en aval et développer une fixation sur les méthodes plutôt que de coordonner son action pour changer les choses.

Bien entendu, nous avons repéré plusieurs bons exemples de réflexion attentive et critique par des auteurs de la RCSP sur les questions économiques. L’apparition, au milieu des années 1990, du discours sur les déterminants socioéconomiques de la santé en amont a donné du poids aux critiques de la politique économique libérale dans une perspective de santé publique. Il faut renforcer cet élan non seulement en multipliant les critiques conceptuelles du libéralisme économique dans les pages de la RCSP, mais au moyen d’études empiriques qui explorent le lien entre les décisions de principe en matière économique et les résultats cliniques à l’échelle des populations. Globalement, nous soutenons, comme d’autres l’ont fait avant nous, que la domination continue du libéralisme et du résidualisme économiques implicites et explicites en santé publique, qui est indissolublement liée aux origines médicales du domaine et qui se manifeste, par exemple, par une interprétation réductrice et sous-théorisée du lien entre le revenu et la santé (Raphael et al., 2006), entrave la capacité de notre communauté de travailler de façon cohérente à la réalisation de ses objectifs déclarés, le bien-être des populations et l’équité en santé, lesquels sont fondamentalement ancrés dans les systèmes économiques et autres. Comme il a été dit ailleurs dans la Revue, il faut d’abord mettre de l’ordre dans nos propres affaires (McLaren & Hancock, 2019). Pour faire un pas dans cette direction, nous recommandons avec enthousiasme l’exercice très instructif de lire les anciens numéros de la Revue.

Lindsay McLaren, Rédactrice scientifique adjointe, RCSP

Eric Mykhalovskiy, Rédacteur associé, CJPH

## Data Availability

Not applicable.

## Footnotes

1 For example, some of the earliest issues of the journal (circa 1910) specify concern with “general prophylaxis” and “social well-being” (personal communication, Ian Culbert, CPHA Executive Director, June 3, 2024; we are grateful to Ian for providing this information).

2 The journal name was *The Public Health Journal* (1910–1928); *The Canadian Public Health Journal* (1929–1942); and the *Canadian Journal of Public Health* (1943–present).

3 The six historical CJPH articles chosen to feature in this special section are republished separately and will be free access for 60 days after publication, after which time they can be accessed (via subscription) through JSTOR (https://www.jstor.org).

4 For a broader historical discussion of eugenics, racism, and other forms of discrimination in Canadian public health, see McLaren (1990) and Valverde (2008).

5 In 1957, the federal government passed the Hospital Insurance and Diagnostic Services Act, a key step in the establishment of Medicare in Canada. The Act set out cost-sharing arrangements between the federal government and the provinces and territories for a publicly administered and funded plan covering a specified group of “inpatient hospital and diagnostic services” (Government of Canada, n.d.).

6 Par exemple, certains des premiers numéros de la Revue (vers 1910) traitent en particulier de « prophylaxie générale » et de « bien-être social » (communication personnelle, Ian Culbert, directeur général de l’ACSP, 3 juin 2024; merci à Ian pour cette information).

7 La Revue s’est successivement appelée *The Public Health Journal* (1910‒1928), la *Revue canadienne d’hygiène publique* (1929‒1942) et la *Revue canadienne de santé publique* (depuis 1943).

8 Les six articles passés de la RCSP choisis pour cette section spéciale sont republiés séparément et seront librement accessibles pendant 60 jours, après quoi ils pourront être consultés (par abonnement) dans la bibliothèque numérique JSTOR (https://www.jstor.org).

9 McLaren (1990) et Valverde (2008) présentent une analyse historique plus large de l’eugénisme, du racisme et d’autres formes de discrimination dans la communauté de la santé publique canadienne.

10 En 1957, le gouvernement fédéral adopte la *Loi sur l’assurance-hospitalisation et les services diagnostiques*, un jalon important dans l’établissement du régime d’assurance-maladie au Canada. La Loi établit entre le gouvernement fédéral et les provinces et territoires des accords de partage des coûts d’un régime géré et financé par le secteur public qui couvre un groupe particulier de « services aux malades hospitalisés » et de « services diagnostiques ». (Government of Canada, n.d.).
